# Should asymptomatic contralateral inguinal hernia be laparoscopically repaired in the adult population as benefits greatly outweigh risks? A systematic review and meta-analysis

**DOI:** 10.1007/s10029-022-02611-z

**Published:** 2022-04-18

**Authors:** Jung B Park, Darren C. Chong, Jessica L. Reid, Suzanne. Edwards, Guy J. Maddern

**Affiliations:** 1 Faculty of Health and Medical Sciences, University of Adelaide, Adelaide, SA 5000 Australia; 2 The Queen Elizabeth Hospital, Woodville, SA 5011 Australia

**Keywords:** Inguinal hernia, Occult, Contralateral, Laparoscopic repair

## Abstract

**Purpose:**

When laparoscopically repairing a symptomatic inguinal hernia, surgeons will discover a contralateral asymptomatic hernia in 22% of patients. It is estimated 30% of asymptomatic hernias become symptomatic and require repair. Thus, should they be repaired in a 2-for-1 operation? The main purpose is to examine the evidence and make a recommendation for the need to repair the contralateral asymptomatic inguinal hernia prophylactically in the adult population during unilateral inguinal hernia presentation.

**Method:**

A systematic literature search was conducted up to 15 February 2021 using PubMed and the Cochrane Library. Management pathway taken, mean operating time, duration of follow-up, pain, duration of hospital stay and perioperative complications were extracted. Risk of bias was assessed using the ROBINS-I tool.

**Results:**

Six non-randomised studies (1774 patients) were included; 978 patients had both hernias repaired, 796 patients had only the symptomatic hernia repaired. There was no significant difference in length of hospital stay, return to activities of daily living nor complications. Mean operating time was slightly lower for patients who had unilateral hernia repair (mean difference = − 14.57 min, 95%CI − 25.59, − 3.45). Reported pain scores were lower for patients who only had one hernia repaired (− 0.33 units, 95%CI − 0.48, − 0.18). The overall risk of bias for the six studies were low-to-moderate risk.

**Conclusion:**

Asymptomatic inguinal hernias can be repaired when found. While there is minimal increase in operation time and pain, no significant difference to total hospital stay. Importantly, this is likely to prevent the need for another operation in almost a third of patients.

**Supplementary Information:**

The online version contains supplementary material available at 10.1007/s10029-022-02611-z.

## Introduction

Laparoscopic inguinal hernia repair is a common general surgery procedure that is on the rise. In Australia, approximately 40,000 hernias were repaired between 2010 and 2011, and 23% were repaired laparoscopically [1]. The total number of laparoscopic hernia repair procedures increased by twofold between 2003 and 2012 [1]. The prevalence of asymptomatic contralateral inguinal hernia in patients who presented with unilateral inguinal hernia is 13%, of which 97% are male [2]. It is estimated that 30% of asymptomatic (including occult) hernias will become symptomatic and will require a second operation [3]. Despite the high prevalence, there is currently no consensus on timing of laparoscopically repair asymptomatic contralateral inguinal hernias. Moreover, there was also controversy on whether to explore the asymptomatic contralateral hernia as per the European Hernia Society (EHS) recommended against routine exploration by TEP, whereas Chao-Chuan Wu et al. study supported simultaneous exploration to prevent contralateral metachronous inguinal hernia [3]. Thus, our aim of this systematic review is to provide a guide on whether contralateral asymptomatic inguinal hernia should be repaired if they were explored during a unilateral laparoscopic repair.

The objective of this systematic review is to evaluate patient outcomes associated with prophylactic contralateral laparoscopic inguinal hernia repair in the adult population who present with a symptomatic unilateral inguinal hernia.

## Methods

This systematic review was produced in accordance with the Preferred Reporting Items for Systematic Reviews and Meta-Analyses (PRISMA) checklist [4]. The systematic review was also registered on PROSPERO to avoid any duplication with other researchers.

### Eligibility criteria

This inclusion criteria for this review were defined as priori (Table 1). Patients with asymptomatic contralateral direct/indirect inguinal hernia, above 18 years of age, with any comorbidities were included. There was no differentiation made for patients who were treated with either totally extraperitoneal (TEP) or transabdominal preperitoneal (TAPP) as this was not within the scope of this study. Prophylactic contralateral inguinal hernia repair was compared against no prophylactic inguinal hernia repair. Relevant outcomes included duration of hospital stay, operating time, time taken to return to activities of daily living (ADL), postoperative pain measured on a visual analogue scale (VAS), intraoperative and postoperative complications. Randomised controlled trials (RCTs) and non-randomised comparative studies were eligible for inclusion.

**Table 1:** PICO criteria

1. Population -Adult population with a unilateral symptomatic inguinal hernia
2. Intervention (I) of interest: -Prophylactic inguinal hernia repair on contralateral side
3. Comparator (C) of interest: -Watchful waiting
4. Outcome Measures (O) of interest: -Duration of hospital stay, mean operating time, time taken to return to ADLs, VAS pain score and percentage of complications

Exclusion criteria included children under 18-year-old, recurrent hernia and other types of hernia except for inguinal hernia. Non-English articles, case reports and systematic reviews were excluded. Studies published prior to the year 2000 were excluded as surgical intervention and patient management methods would be outdated due to the development of technology that would affect the outcome of this study, particularly in regard to patient recovery.

### Information sources and search strategy

Two databases (PubMed and the Cochrane Library) were searched up to 15th of February 2021. No search limits or filters were used. Grey literature was not sought. The following search strategy was used, combining keywords and medical subject headings:

("hernia, inguinal"[MeSH Terms] OR "inguinal hernia*"[Title/Abstract] OR "groin hernia*"[Title/Abstract] OR "contralateral*"[Title/Abstract] OR "bilateral*"[Title/Abstract]) AND ("Repair"[Title/Abstract] OR "herniorrhaphy"[MeSH Terms] OR "herniorrhaphy"[Title/Abstract] OR "laparoscopy"[MeSH Terms] OR "laparoscopy*"[Title/Abstract] OR "hernioplasty*"[Title/Abstract]) AND ("risk factors"[MeSH Terms] OR "disease progression"[MeSH Terms] OR "watchful waiting"[MeSH Terms] OR "recurrence"[MeSH Terms] OR "complication*"[Title/Abstract] OR "prognosis*"[Title/Abstract]) AND ("adult"[MeSH Terms] OR "adult*"[Title/Abstract]).

### Study selection

Initially, studies were screened and included based on title and abstract executed by two authors (D.C & J.P) independently. Potentially relevant studies were then evaluated based on full text to determine if outcomes relevant to this review were reported. Disagreements regarding study selection were settled via consensus.

### Data extraction and analysis

Relevant data were extracted into a predefined template. The following data were extracted: type of study, year of publication, number of participants, management pathway taken (unilateral or bilateral repair), mean operating time, duration of follow-up and outcome of management pathway (pain, duration of hospital stay, intraoperative and postoperative complications).

Data analyses were performed using Stata Statistical Software (Release 15.1 College Station, TX: StataCorp LP). In this meta-analysis, continuous outcomes were evaluated as mean differences with 95% confidence intervals (CIs), and dichotomous outcomes were evaluated as odds ratios with 95% CIs. Meta-analysis was performed for outcomes that were reported by at least two studies, which reported sufficient detail (e.g. n, mean and standard deviation in unilateral and bilateral groups). The *I*^2^ statistic was used to evaluate heterogeneity (with *I*^2^ > 50% indicating substantial heterogeneity) as was Cochran’s *Q* (with *p* value < 0.05 indicating significant heterogeneity) [5]. In view of the heterogeneity found for a number of the variables in this meta-analysis, a random-effects model using the method of DerSimonian & Laird with estimate of heterogeneity taken from Mantel–Haenszel model used throughout. A *p* value of < 0.05 denoted statistical significance. Publication bias was not measured quantitatively due to lack of applicable studies. Publication bias tests such as funnel plot asymmetry were used when there were more than ten studies included in the meta-analysis, as when it is less than ten studies, the power of the test is low [5].

### Risk of bias

The risk of bias in the included studies was evaluated using the Risk of Bias in Non-randomised Studies (ROBINS-I) tool developed by the Cochrane collaboration. ROBINS-I tool was used as it is designed to measure risk of bias in non-randomised studies. Signalling questions were used to judge the risk of bias across seven domains (i.e. confounding, selection of participants into the study, classification of intervention/post-intervention, deviations from the intended interventions, missing data, measurement of outcomes and selection of the reported result). Both authors (D.C & J.P) assessed the risk of bias independently in each study. Discrepancies between reviewers were resolved via consensus.

## Result

### Study selection and characteristics

**Fig. 1:**
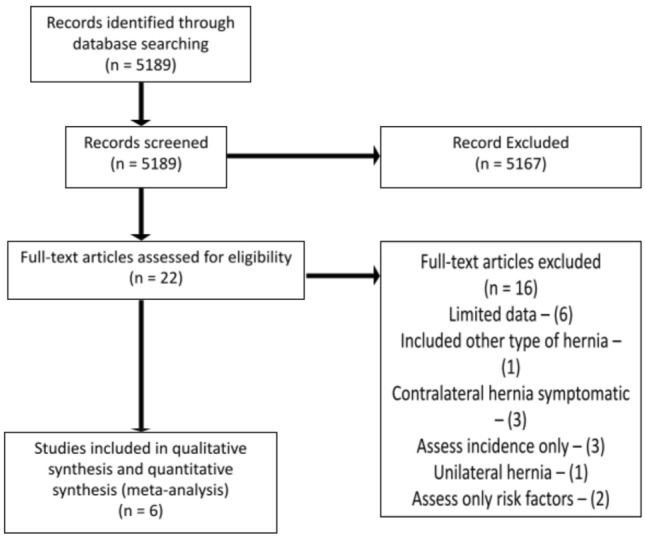
PRISMA flow diagram

The results of the study selection process are reported in Fig. 1. The search strategy resulted in 5189 studies reviewed by title and abstract, 22 studies were selected for full-text review, and 6 studies were eligible for inclusion in the qualitative synthesis and meta-analysis.

Three of these papers were retrospective and three were prospective. All were non-randomised. Three studies [6–8] had no female patients while the rest of the studies had a majority of male patients [9–11] of a mean age of 45.7 (range 14–73) years. The studies followed-up patients for a mean duration of 47.0 (range 12–84) months.

A total of 1749 patients with inguinal hernia procedures were included; 962 (55%) patients had both hernias repaired, while 787 (45%) patients had only the symptomatic hernia repaired. Two studies included patient group with clinically symptomatic bilateral hernia, therefore, a total of 103 patients from the two studies have been excluded from this analysis by the authors. The studies were included in the review as majority of the patients had asymptomatic contralateral inguinal hernia. Four studies included the type of inguinal hernia for the asymptomatic contralateral inguinal hernia. 92% of asymptomatic contralateral inguinal hernias were indirect inguinal hernia [6, 8, 10, 11]. Three studies noted which side was the contralateral inguinal hernia, 62% of asymptomatic contralateral inguinal hernias are located on the left side with the right side being symptomatic (Table 2) [10].

### Risk of bias

**Fig. 2:**
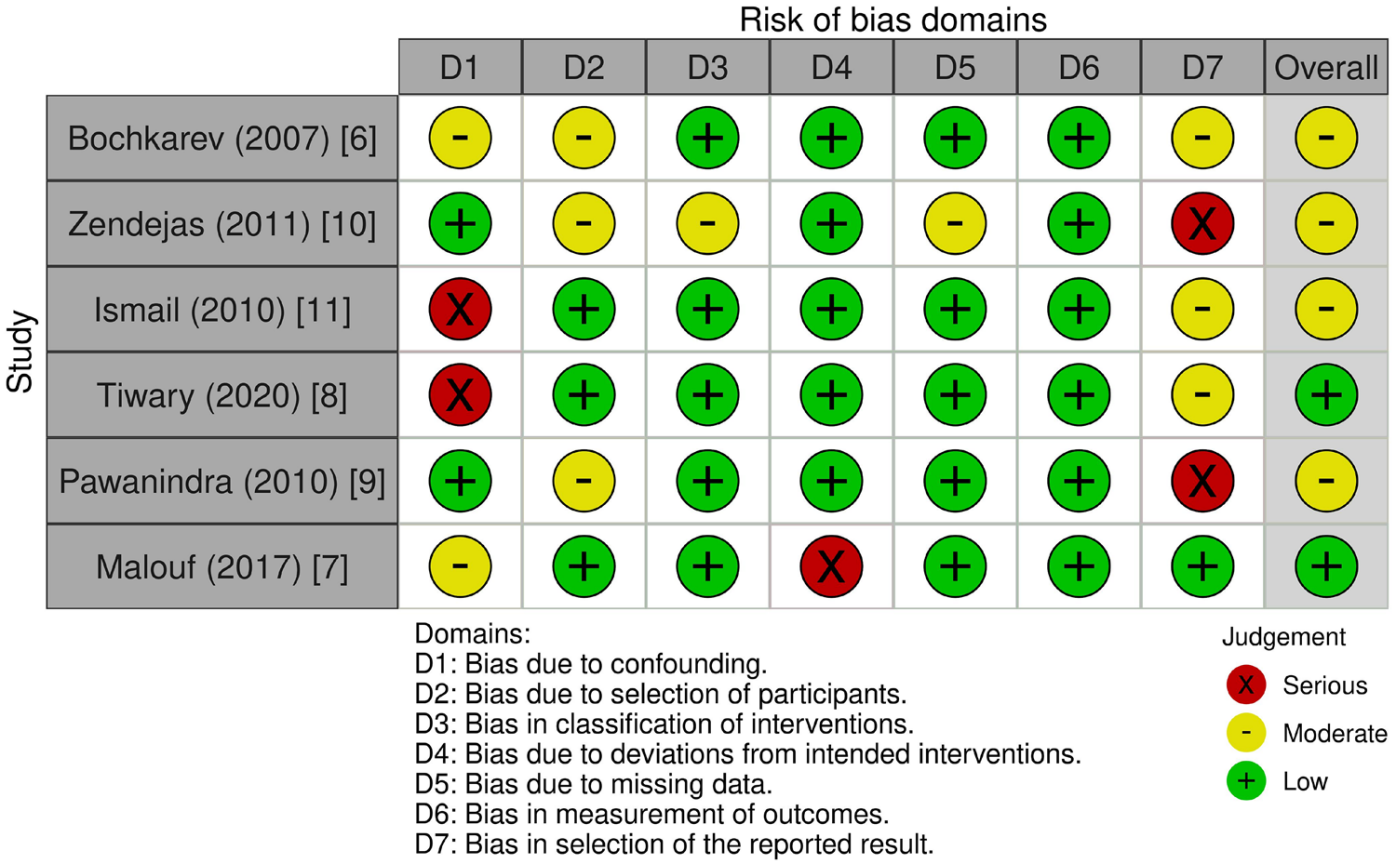
Risk of bias for individual studies

The overall risk of bias was low-to-moderate risk, with most studies at low risk of bias due to classification of interventions, deviations from intended interventions, missing data and measurement of outcomes. There was a low-to-serious risk of bias due to the presence of numerous unidentified confounding factors which might affect the result of the study [8, 9]. The risk of bias regarding selection of the reported results was also serious, as some omitted measurement instruments in a study, multiple statistical analyses was performed, or consisted of multiple subgroups with more detailed reporting (Fig. 2) [10, 11].

**Table 2:** Study selection and characteristics

Study	Study type	Country	Recruitment periods	Sex (M/F)	Mean age (years)		Follow-up period (months)	
					Repaired	Not repaired	Repaired	Not repaired
Bochkarev (2007) [6]	Prospective Non-Randomised Concurrent Control Study	USA	Not reported but 48 months duration	100/0	48 (median)		4–46	
Zendejas (2011) [11]	Retrospective Non-Randomised Concurrent Control Study	USA	September 1995–December 2009	397/12	52.5		0–168	
Ismail (2010) [9]	Retrospective Non-Randomised Concurrent Control Study	India	January 2005–December 2007	919/10	46.3	45	12–40	
Tiwary (2020) [8]	Prospective Non-Randomised Concurrent Control Study	India	August 2017–July 2019	30/0	40.5		12	
Pawanindra (2010) [10]	Prospective Non-Randomised Non-Concurrent Control Study	India	March 2003–March 2007	150/0	36.64	37.16	60 – 72	72–84
Malouf (2017) [7]	Retrospective Non-Randomised Concurrent Control Study	Australia	July 2011–November 2015	234/0	47	52	84

### Duration of hospital stay

**Fig. 3:**
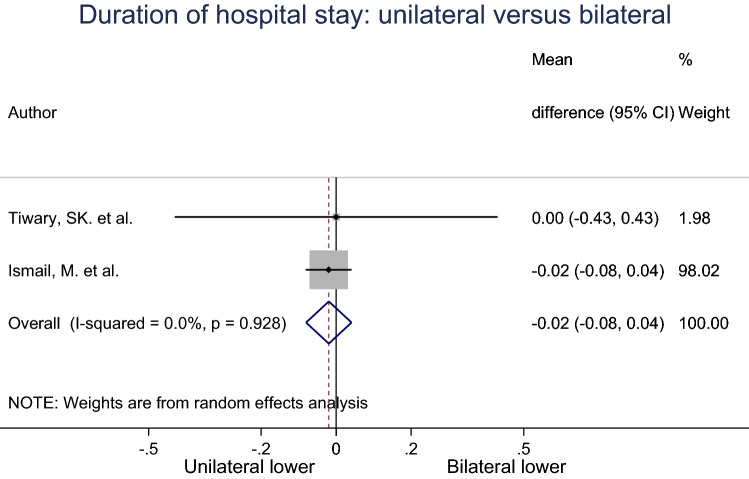
Meta-analysis of mean duration of hospital stay for unilateral compared to bilateral inguinal hernia patients

The mean duration of hospital stay for unilateral and bilateral groups were pooled across two studies using a random effects meta-analysis (Fig. 3). Heterogeneity in the study estimates was assessed using the *I*-squared statistic (92.8%) and Cochran’s *Q* *P* value (< 0.0001) which showed substantial heterogeneity. There is no difference between unilateral and bilateral in duration of hospital stay. This result was not statistically significant. 98% of the weight of this analysis came from Ismail et al. The studies included in analysis had an overall low risk of bias.

### Operating time

**Fig. 4:**
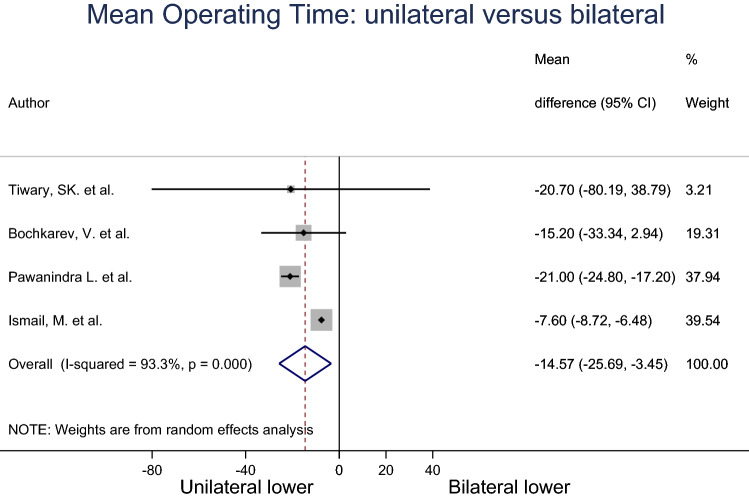
Meta-analysis of mean operating time for unilateral compared to bilateral inguinal hernia patients

The mean operating time for unilateral and bilateral groups were pooled across four studies using a random effects meta-analysis model (Fig. 4). Heterogeneity in the study estimates was assessed using the *I*-squared statistic (93.3%) and Cochran’s *Q* *P* value (< 0.0001) which showed much heterogeneity. The overall mean operating time in unilateral group was 14.57 min less compared to bilateral group, and this result was statistically significant (95% Cl − 25.59, − 3.45). The studies included in this analysis had an overall of low-to-moderate risk of bias.

### Return to activities of daily living (ADL)

**Fig. 5:**
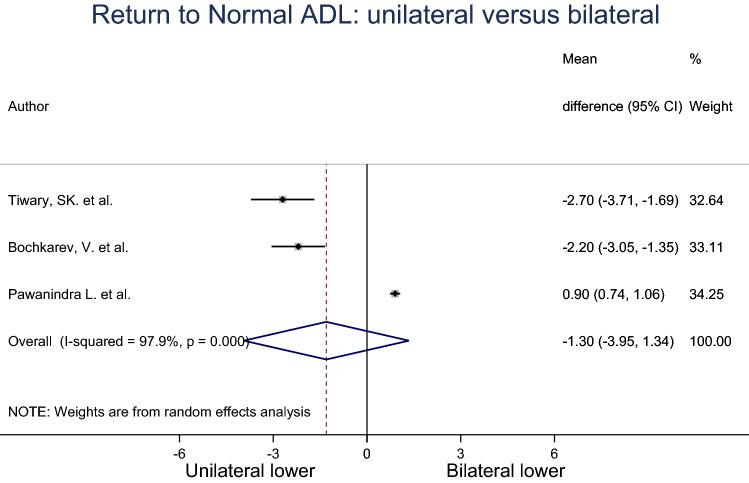
Meta-analysis of return to normal ADL for unilateral compared to bilateral inguinal hernia patients

The mean time to return to normal ADL for unilateral and bilateral groups were pooled across three studies using a random effects meta-analysis model (Fig. 5). Heterogeneity in the study estimates was assessed using the *I*-squared statistic (97.9%) and Cochran’s *Q* *P* value (< 0.0001). The mean return to normal ADL in the unilateral group was 1.30 units less compared to the bilateral group, but this result was not statistically significant (95% Cl − 3.95, 1.34). The studies included in this analysis had an overall moderate risk of bias.

### VAS pain score

**Fig. 6:**
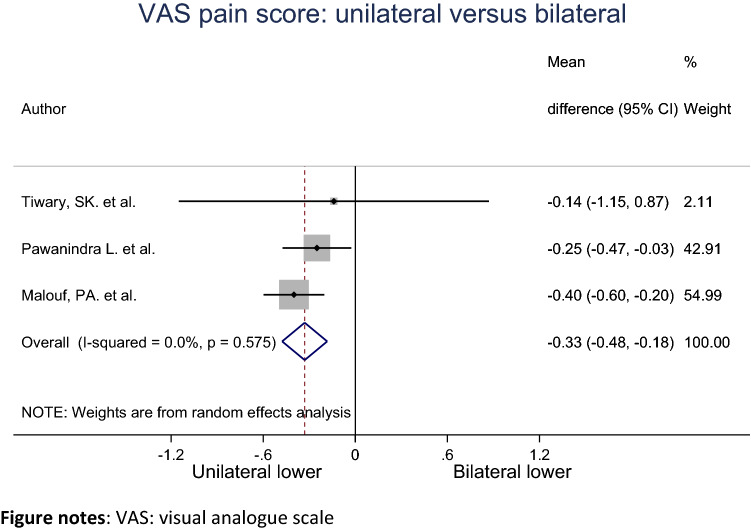
Meta-analysis of VAS pain score for unilateral compared to bilateral inguinal hernia patients. *VAS* visual analogue scale

The mean VAS pain score for unilateral and bilateral groups were pooled across three studies using a random effects meta-analysis model (Fig. 6). Heterogeneity in the study estimates was assessed using the *I*-squared statistic (0.0%) and Cochran’s *Q* *P* value (0.575) which showed no heterogeneity. The mean VAS pain score in the unilateral group was 0.33 units less compared to the bilateral group, and the result is statistically significant (95% Cl − 0.48, − 0.18). The studies included in this analysis had an overall low risk of bias.

### Percentage complications

**Fig. 7:**
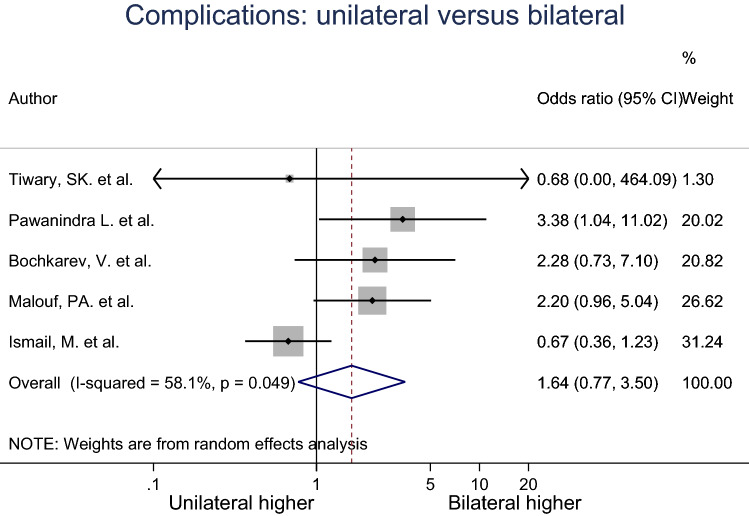
Meta-analysis of complications for unilateral compared to bilateral inguinal hernia patients

Log Odds Ratio (OR) was calculated for the percentage of complications for unilateral and bilateral groups, as well as the standard error of the log OR. The log ORs were pooled across five studies using a random effects meta-analysis model (Fig. 7). Heterogeneity in the study estimates was assessed using the I-squared statistic (58.1%) and Cochran’s *Q* *P* value (< 0.049) which showed slight heterogeneity. However, because a random effects model was used, the degree of heterogeneity is not relevant. The odds of having complications from surgeries in the unilateral group was 1.84 times less compared to the bilateral group, but this result was not statistically significant (95% Cl 0.77, 3.50). The studies included in this analysis had an overall moderate risk of bias.

## Discussion

Six studies were included in this meta-analysis. There were two patient groups in each study: patients who had a ‘bilateral’ repair (both symptomatic side and asymptomatic contralateral side) and those who were treated for ‘unilateral’ hernia repair; thus, the asymptomatic contralateral hernia was left alone. All surgical interventions were done laparoscopically, no open surgery repairs were performed in any of the trials. Furthermore, we understood and agreed that exploration of the contralateral side using TEP is not recommended as per the European Hernia Society. Thus, all investigation of asymptomatic inguinal hernia will need to be diagnostic laparoscopy, though we acknowledge the possibility that diagnostic laparoscopy may miss a contralateral hernia.

According to Malouf’s study, bilateral inguinal hernia repair can be performed with no increased morbidity and has only a very temporary impairment in terms of quality of life compared to unilateral inguinal hernia repair [7]. While the estimate rate of conversion from asymptomatic to symptomatic is approximately 1.2% per year, factors such as age, comorbidities and poor nutritional status can increase this risk [11, 12]. This includes occult inguinal hernia, which is an asymptomatic hernia not detectable by physical examination.

Our analysis reported the mean operating time for unilateral repair was shorter by an average of 14.57 min (95% CI − 22.16, − 10.59) compared to bilateral. It is not surprising that operating time for bilateral hernia repair would take longer than the unilateral hernia repair. Longer operating time would lead to an increase in cost. Thumbe et al. reported that the mean total cost of a laparoscopic hernia repair is GBP 1074.00, while the cost of repairing the contralateral hernia at the same time was slightly higher due to the price of the mesh [13]. Therefore, we believe that to prevent a second operation that increases the cost for the health service and puts the patient at risk of adverse effects from anaesthesia for a second time, asymptomatic contralateral inguinal hernia should be considered.

We found that patients who underwent repair for contralateral inguinal hernia had a statistically significant higher VAS pain score (0.33). However, as the scale ranges from 1 to 10, we do not believe this increase is clinically significant as it is only a very slight increase. Furthermore, despite VAS pain score being higher in the bilateral group, O’Dwyer et al. argued that operation of an asymptomatic hernia has little effect on the rate of long-term chronic pain for patients [14]. This is also supported by Malouf’s study as the VAS score was 0.46 units (Cl − 0.096, 0.0046) higher for bilateral repair compared to unilateral at 2 weeks postoperative but this difference resolved by 6 weeks [7]. In addition, although the long-term quality of life was not studied in this review as patients were not followed-up, Malouf’s study showed that there is no difference at 6 weeks between unilateral and bilateral repair [7].

Although the mean duration of hospital stay, mean return to ADL and the percentage of postoperative complications did have some difference in scores, these were not statistically significant. Kockerling et al. argued that there is a significantly higher risk of having postoperative urinary bladder injury in bilateral repair compared to unilateral repair [15]. Kockerling et al. also commented that although bilateral inguinal hernia repair in a single operation result in an even higher complication rate, repairing bilateral inguinal hernia in two separate operations will also result in a higher complication rate. On the other hand, numerous studies argued that there is no increase in the rate of surgical complications and morbidity in bilateral inguinal hernia repair compared to unilateral inguinal hernia repair [7, 10, 11, 16]. More data and analysis will need to be done specifically in that area to clarify the issue.

Limitations of this meta-analysis include the small number of studies in our review. All studies are prospective or retrospective studies with no randomised controlled studies. These factors led to an insufficient level of evidence. However, we applied the ROBINS-I tool to identify any confounding factors and evaluate the quality of these studies. Furthermore, majority of the studies did not report essential data regarding the type of hernia, type of mesh used, comorbidities patients had prior to surgery, previous surgical history, patient’s risk factors and therefore these variables were not included as part of our study analysis. In addition, the post-operative complications in the six studies reviewed were not standardised and were highly variable in each study, which may affect our meta-analysis result. Furthermore, in duration of hospital stay, despite it being not statistically significant, 98.02% of the weight is from Ismail et al. study (Fig. 3). This made the analysis mirror the finding of the study. Lastly, the studies had a short follow-up period, this led to the inability to clearly investigate the rate of long-term complications and conversion from asymptomatic to symptomatic inguinal hernia postoperatively as it would provide a better indication for bilateral hernia repair prophylactically.

## Conclusion

The treatment of an asymptomatic contralateral inguinal hernia has been a debate in the past decade with no consensus. This is because that majority (70%) of inguinal hernias diagnosed laparoscopically never become symptomatic thus no treatment required. Hence, we aimed to assess the risks and benefits to determine if it is necessary to repair asymptomatic contralateral inguinal hernia prophylactically. Our analysis indicates that, repairing the asymptomatic contralateral inguinal hernia is associated with more benefit than risk, therefore, the repair of the asymptomatic hernia could be considered. There is likely to be a small increase in operation time and pain when performing a prophylactic repair of asymptomatic contralateral inguinal hernia compared to just repairing a symptomatic inguinal hernia. Patient’s perception of pain and increased cost for prolonged duration of surgery cannot be ignored. However, there is no significant difference to total hospital stay, postoperative complications or duration for returning to ADL. Therefore, the most significant factor that would prevent patients from undergoing prophylactic inguinal hernia repair would be the higher risk of developing post-operative pain at the surgical site. This indicates that although there may be immediate risk of increased pain, in the long term, prophylactic repair may prevent repeat procedures with no significant side effects of patient recovery to daily life. Despite these being significant findings, there is also possibility of having a high risk of bias for confounding as these findings were informed by observational data and the lack of evidence in the terms of the population at risk of developing a symptomatic hernia shows that further analyses will need to be done to answer this fundamental question. Finally, we believe it is only ethically appropriate that patients should have this option discussed preoperatively and consent obtained before proceeding with the repair especially in an elective surgery setting.

## Supplementary Information

Below is the link to the electronic supplementary material.

Supplementary file1 (PDF 168 KB)
